# Development and Validation of an Immune-Related Prognostic Signature in Cervical Cancer

**DOI:** 10.3389/fonc.2022.861392

**Published:** 2022-05-16

**Authors:** Rongjia Su, Chengwen Jin, Hualei Bu, Jiangdong Xiang, Lina Zhou, Chengjuan Jin

**Affiliations:** ^1^Department of Obstetrics and Gynecology, Shanghai General Hospital, Shanghai Jiaotong University School of Medicine, Shanghai, China; ^2^Department of Gynecologic Oncology, International Peace Maternity and Child Health Hospital, Shanghai Jiaotong University School of Medicine, Shanghai, China; ^3^Department of Central Laboratory, Shanghai Chest Hospital, Shanghai Jiaotong University School of Medicine, Shanghai, China; ^4^Department of Obstetrics and Gynecology, Qilu Hospital, Shandong University, Jinan, China

**Keywords:** cervical cancer, weighted gene co-expression network analysis (WGCNA), least absolute shrinkage and selection operator (LASSO), prognostic signature, immune signature, gene set enrichment analysis (GSEA)

## Abstract

**Background:**

Cervical cancer is the fourth most frequent gynecological malignancy across the world. Immunotherapies have proved to improve prognosis of cervical cancer. However, few studies on immune-related prognostic signature had been reported in cervical cancer.

**Methods:**

Raw data and clinical information of cervical cancer samples were downloaded from TCGA and UCSC Xena website. Immunophenoscore of immune infiltration cells in cervical cancer samples was calculated through the ssGSEA method using GSVA package. WGCNA, Cox regression analysis, LASSO analysis, and GSEA analysis were performed to classify cervical cancer prognosis and explore the biological signaling pathway.

**Results:**

There were eight immune infiltration cells associated with prognosis of cervical cancer. Through WGCNA, 153 genes from 402 immune-related genes were significantly correlated with prognosis of cervical cancer. A 15-gene signature demonstrated powerful predictive ability in prognosis of cervical cancer. GSEA analysis showed multiple signaling pathways containing Programmed cell death ligand-1 (PD-L1) expression and PD-1 checkpoint pathway differences between high-risk and low-risk groups. Furthermore, the 15-gene signature was associated with multiple immune cells and immune infiltration in tumor microenvironment.

**Conclusion:**

The 15-gene signature is an effective potential prognostic classifier in the immunotherapies and surveillance of cervical cancer.

## Introduction

According to estimates from GLOBOCAN 2018, cervical cancer was the fourth most common cancer among women, with approximately 570,000 new cases and 311,000 deaths (1). Cure rate for earliest stages is more than 90%, whereas locally advanced lesions treated with multimodality therapy can only achieve 65% cure rate in stage II lesions and 55% in stage III lesions (2). Therapies to improve survivorship are in desperate need for cervical cancer especially locally advanced disease.

With rapid development of precision medicines, novel therapeutic strategies, especially immunotherapies, have been proposed to significantly improve clinical outcomes of cervical cancer (3, 4). Harnessing an antitumor immune response has been a fundamental strategy in cancer immunotherapy. A paradigm shift has appeared in cancer immunotherapy: from traditional immune enhancement with low-objective responses and frequent adverse events to more effective and less toxic reactions immune normalization (5, 6).

Cancer immunotherapies, such as administration of the cytokine Interleukin-2 (IL-2), adoptive cell transfer, and the checkpoint modulators CTLA-4 and PD-1, have proved effective in clinical practice (6). Blockade of the checkpoint modulators cytotoxic T-lymphocyte-associated antigen 4 (CTLA-4) and programmed cell death protein-1 (PD-1) starts the field of immune normalization in immunotherapy. Upregulated PD-1 in tumor microenvironment inhibits an effector T cell antitumor immune response, and therapies blocking this pathway have proven effective against multiple tumor types (5). Anti-PD therapy perform antitumor immunity mainly through the following three principles:1) targeting a tumor-induced immune escape mechanism, 2) selectively modulating immunity in the tumor microenvironment, and 3) resetting immunity in the tumor microenvironment (5). It has been proved that patients with high mutation burden and burden of potential neoepitopes benefit more from immunological checkpoint blockade (7–11). Immunotherapy plays a dispensable role in management of cervical cancer. In KEYNOTE-158, pembrolizumab has been approved by the US Food and Drug Administration for use in advanced cervical cancer with progressive disease either during or after chemotherapy (12). The objective response rate was 26.3% with a disease control rate of 68.4% for immunotherapy in cervical cancer (4). However, only small proportion patients benefited from immunotherapy, and this proportion will hopefully increase with better patient selection and combination therapy. Hence, it is urgent to find potential biomarkers for prediction of response to checkpoint immunotherapy and the rationale for the use of checkpoint immunotherapy.

In the present study, we qualify immune cells infiltration in cervical cancer and analyze the correlation between immune cells and cancer prognosis. Hub genes regulating prognosis through immune infiltration in cervical cancer were identified by weighted gene co-expression network analysis (WGCNA) and least absolute shrinkage and selection operator (LASSO). It was suggested by infiltrated immune cells and pathway enrichment analysis that our immune-related signature was closely related to tumor prognosis and could predict response of immunotherapy. A robust immune-related prognostic signature based on transcriptomics in cervical cancer was constructed and validated.

## Materials and Methods

### Data Source and Processing

**Figure 1:**
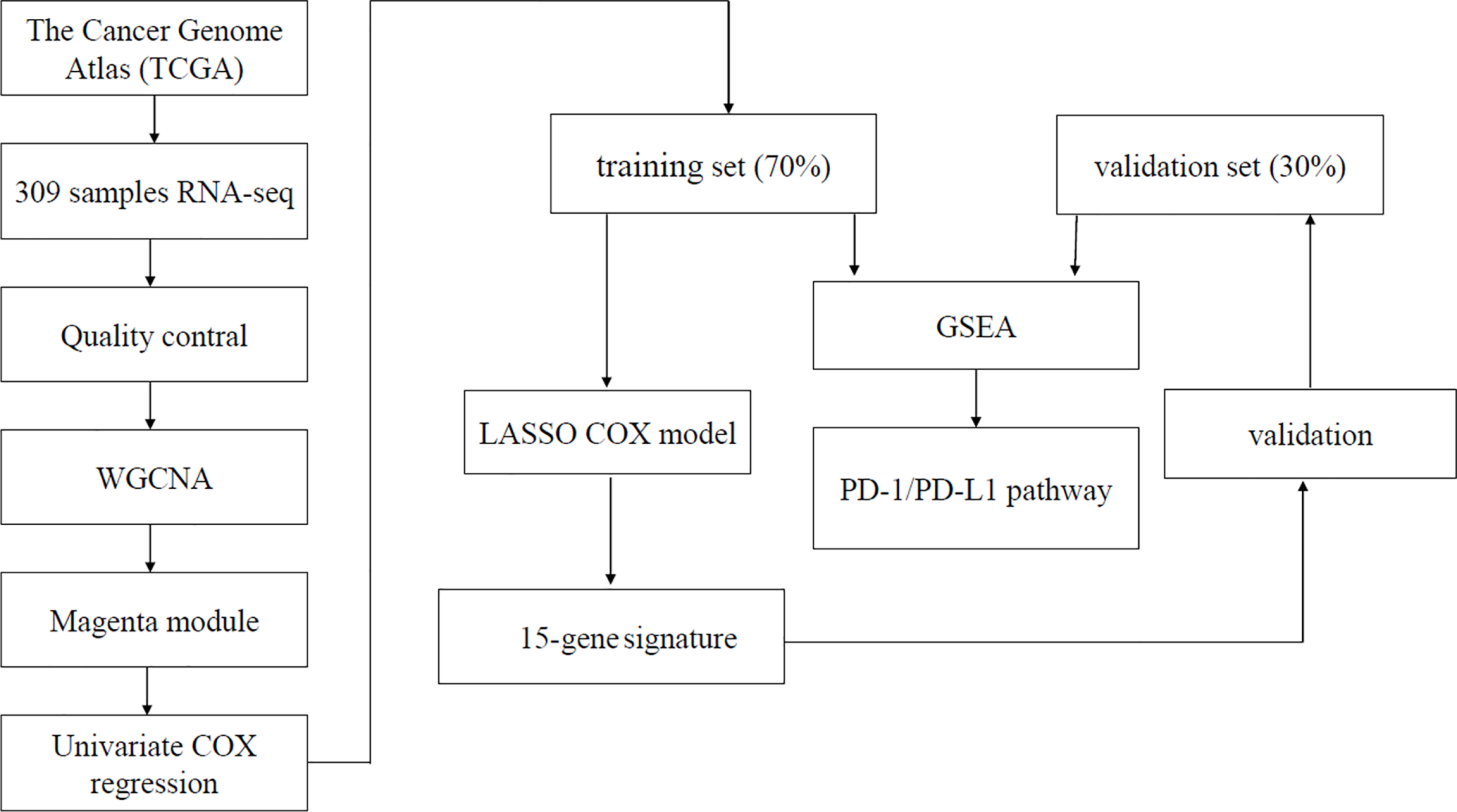
Flowchart of the 15-gene signature prognostic model construction process.

The gene expression profiles and clinical information of cervical cancer were downloaded from The Cancer Genome Atlas (TCGA) Genomic Data Commons Data Portal (https://portal.gdc.cancer.gov/). Patients with pathologically confirmed cervical cancer and complete information about transcriptomics overall survivals (OSs) were included in this study. Finally, a total of 304 primary cervical cancer samples, two metastatic cervical cancer samples, and three normal cervix samples from TCGA were analyzed in our study. Details on clinical information of the included samples were summarized in **Table 1**. A workflow of this study was indicated in **Figure 1**.

**Table 1:** Clinical characteristics of 309 samples from TCGA.

	Alive (N = 235)	Dead (N = 74)	Overall (N = 309)
**Age (years)**			
Mean (SD)	47.4 (13.2)	50.8 (15.4)	48.2 (13.8)
Median [Min, Max]	46.0 [20.0, 88.0]	48.0 [21.0, 79.0]	46.0 [20.0, 88.0]
**Pathology**			
Adenocarcinoma	24 (10.2%)	5 (6.8%)	29 (9.4%)
Adenosquamous carcinoma	4 (1.7%)	1 (1.4%)	5 (1.6%)
Basaloid squamous cell carcinoma	1 (0.4%)	0 (0%)	1 (0.3%)
Endometrioid adenocarcinoma	3 (1.3%)	0 (0%)	3 (1.0%)
Mucinous adenocarcinoma	12 (5.1%)	5 (6.8%)	17 (5.5%)
Papillary squamous cell carcinoma	1 (0.4%)	0 (0%)	1 (0.3%)
Squamous cell carcinoma	190 (80.9%)	63 (85.1%)	253 (81.9%)
**Stage**			
I	125 (53.2%)	38 (51.4%)	163 (52.8%)
II	60 (25.5%)	11 (14.9%)	71 (23.0%)
III	35 (14.9%)	11 (14.9%)	46 (14.9%)
IV	8 (3.4%)	14 (18.9%)	22 (7.1%)
Missing	7 (3.0%)	0 (0%)	7 (2.3%)
**Sample**			
Metastatic	1 (0.4%)	1 (1.4%)	2 (0.6%)
Primary tumor	232 (98.7%)	72 (97.3%)	304 (98.4%)
Solid tissue normal	2 (0.9%)	1 (1.4%)	3 (1.0%)

### Infiltration of Immune Cells

Tumor-infiltrating immune cells can be quantified from RNA sequencing (RNA-seq) data of human tumors using bioinformatics approaches. Single-sample gene set enrichment analysis (ssGSEA) calculated and qualified the infiltration of immune cells through RNA-seq data. The infiltration of 28 immune cells was obtained. Immunophenoscore (normalized enrichment score) of each TCGA cervical cancer sample was calculated through ssGSEA. The ssGSEA ranks the genes by their absolute expression in a sample and computes enrichment score by integrating the differences between the empirical cumulative distribution functions of the gene ranks (13).

### Survival Analysis

Univariate Cox regression analysis was performed to identify the association between cervical cancer survival and immunophenoscore of infiltrated immune cells. Forest plot was drawn to demonstrate the influence of infiltrated immune cells on survival. The best separation statistic was performed by use of “survminer” package that divides gene expression into high groups and low groups based on best separation. Next, Kaplan–Meier curve was made to further analyze relationship between survival and infiltration immune cells.

### Construction of Weighted Gene Co-Expression Network Analysis

WGCNA is an algorithm for finding genetic interactions in a weighted manner. It is used to build a gene co-expression networks to mine network modules closely associated with clinical traits through systematic biological method (14). In this study, immunophenoscore of the infiltrated immune cells was regarded as target clinical traits. Genes expressing not available (NA) were removed. The top 25% genes with most median absolute deviation used as a robust measure of variability were selected for WGCNA analysis (15, 16).

Using the WGCNA function adjacency, an adjacency matrix is constructed by computing the Pearson correlation between all pairs of genes in the selected sample. Genes were divided into different gene modules based on the dissimilarity measure. A hierarchical clustering tree was constructed with different branches of the tree representing different gene modules (15–17).

The WGCNA network was built. There were eight immune cells associated with cervical cancer survival. The magenta module was the module that most related to infiltration of immune cells. A total of 402 genes contained in magenta module were analyzed for survival using the standardized expression data FPKM. Finally, we found that 153 genes were associated with survival (p < 0.05) in magenta module.

### Screening Hub Genes by LASSO and Survival Analysis

The 153 genes with the highest correlation associated with survival were picked out from WGCNA analysis. Then, hub genes were further screened from the 153 genes by the use of LASSO. Survival analysis of hub genes was performed using “survival” and “survminer” packages to verify whether differently expressed genes affected tumor prognosis. Data of cervical cancer from University of California, Santa Cruz (UCSC) Xena were downloaded and applied to compare the expression of hub genes in normal cervix and cervical cancer.

### Construction of Prognostic Scoring Model

Patients with cervical cancer in TCGA were randomly divided into two groups by stage: the training group of 70% and the test group of 30% by the use of caret R package. Finally, a total of 199 patients were used as training group and 74 patients were regarded as test group. The selected key genes for support vector machine analysis were used to fit a LASSO Cox–proportional hazards (Cox-PH) model for selecting an optimal panel of predictive genes with penalized package of R (18). Optimal lambda value was computed through a 10 cross-validations. Next, Cox-PH coefficients and infiltration of immune cells levels of these selected genes were used to calculate prognostic score as follows: Risk Score = Σ (coef_gene_ × immunophenoscore_gene_), where Coef_gene_ and immunophenoscore_gene_ suggest Cox-PH coefficient and immunophenoscore level of a gene, respectively.

### Validation of Prognostic Scoring Model

We calculated risk score of every patient in the training and test group by the model. We separated the training group and test group into a high-risk group and a low-risk group with the median risk score as cutoff, respectively. Kaplan–Meier curve was applied to obtain and compare the OS time of two risk groups. ROC (operating characteristic curve) was performed to evaluate the predictive accuracy of the model.

### Comparison of Eight Immune Cell Subtypes Between High-Risk and Low-Risk Groups

To explore the differences of immune cell subtypes between high-risk and low-risk groups, the eight immune cell subtypes associated with OS in cervical cancer were assessed in test and train cohorts. Mann–Whitney U-test was used to compare differences in immune cell subtypes in the high-risk and low-risk groups.

### Gene Set Enrichment Analysis (GSEA)

All 304 patients with primary cervical cancer were evaluated by prognostic scoring model and then divided into high-risk group and low-risk group based on the standard cutoff. The global gene expression was analyzed and displayed with volcanic maps using the limma package in R. GSEA was conducted respectively to search “all gene sets” enriched in the samples with high-risk group and low-risk group. The differentially expressed genes were enriched in PD-L1 pathway functional sets (19).

## Results

### Quantify Immune Cell Infiltration and Survival Analysis

**Figure 2:**
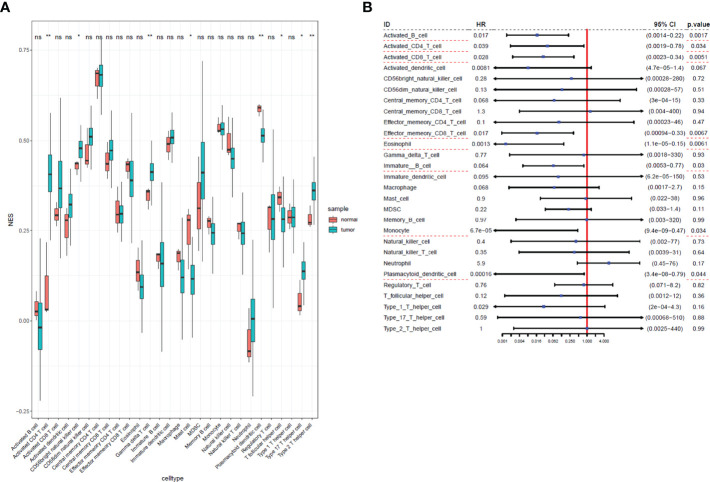
Immunophenoscores and hazard ratios of 28 immune cells in cervical cancer. **(A)** Boxplot of immunophenoscores from 28 immune cells TCGA cervical cancer and normal samples. **(B)** Forest plot of hazard ratios in each infiltrated immune cell for OS. Infiltrated immune cell subtypes significantly associated with overall survival were underlined with a red dashed line. *: P<0.05, **: P<0.01, ns, no significance.

**Figure 3:**
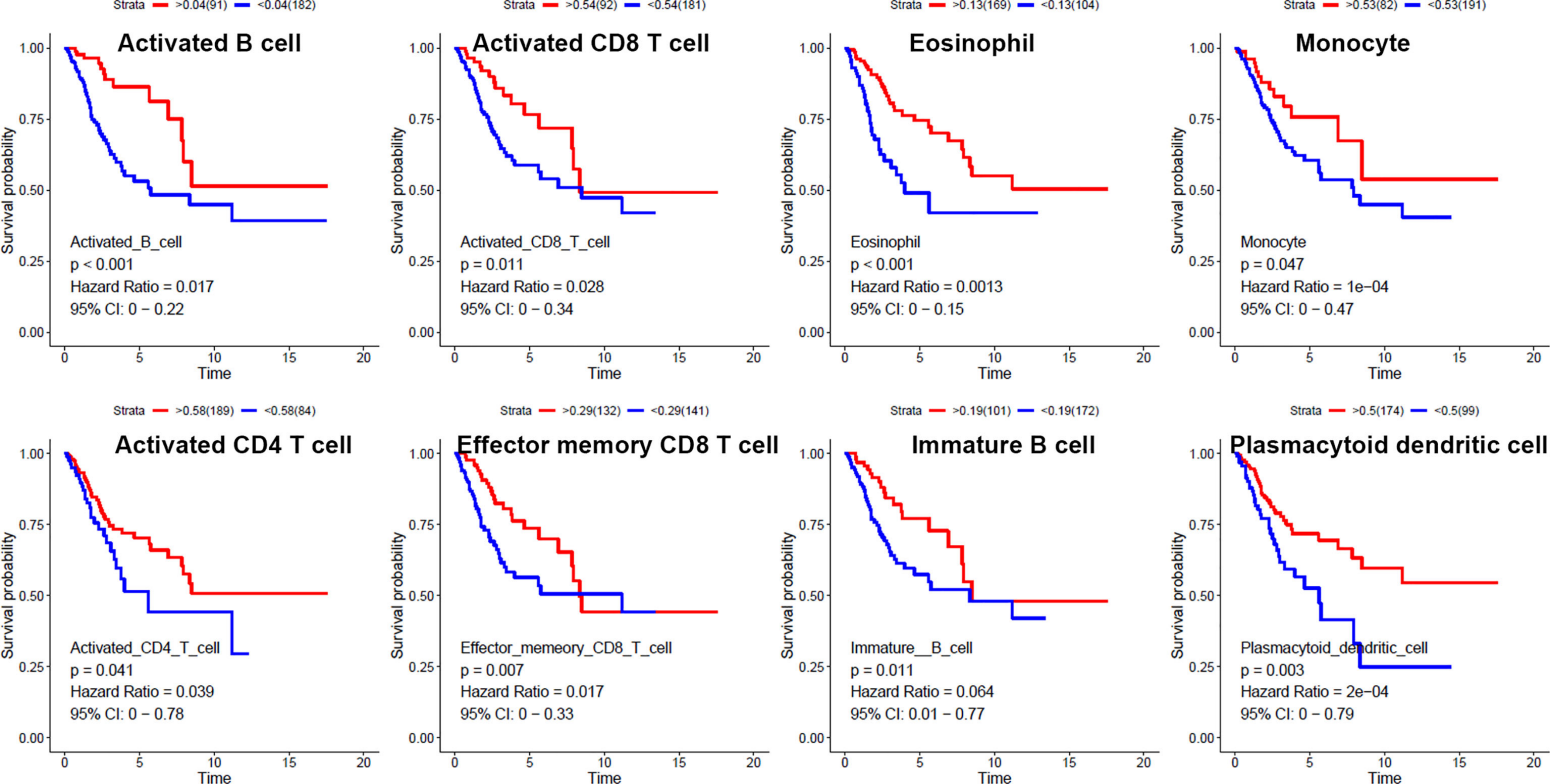
Kaplan–Meier curves for overall survival of eight infiltrated immune cells. Activated B cell, activated CD8 T cell, eosinophil, monocyte, activated CD4 T cell, effector memory CD8 T cell, immature B cell, and plasmacytoid dendritic cell were associated with overall survival of cervical cancer.

We used ssGSEA to quantify mRNA data for immune cell infiltration. Finally, 28 infiltrating immune cells were included. Immunophenoscores of 304 primary cervical cancer samples, two metastatic cervical cancer samples, and three normal cervix samples for 28 immune cells were calculated and demonstrated in **Figure 2A**. Univariate Cox regression analysis was performed to identify association between immune cells and OS of cervical cancer, and the results are shown in **Figure 2B**. It indicated that eight immune cells were correlated with OS significantly. Moreover, these eight immune cells (activated B cell, activated CD8 T cell, eosinophil, monocyte, activated CD4 T cell, effector memory CD8 T cell, immature B cell, and plasmacytoid dendritic cell) were all protective factors for OS. Kaplan–Meier curve validated that high expression of these eight immune cells were related to longer OS time suggested worse prognosis (**Figure 3**).

### The Weighted Gene Co-Expression Network Analysis Construction and Key Module Identification

**Figure 4:**
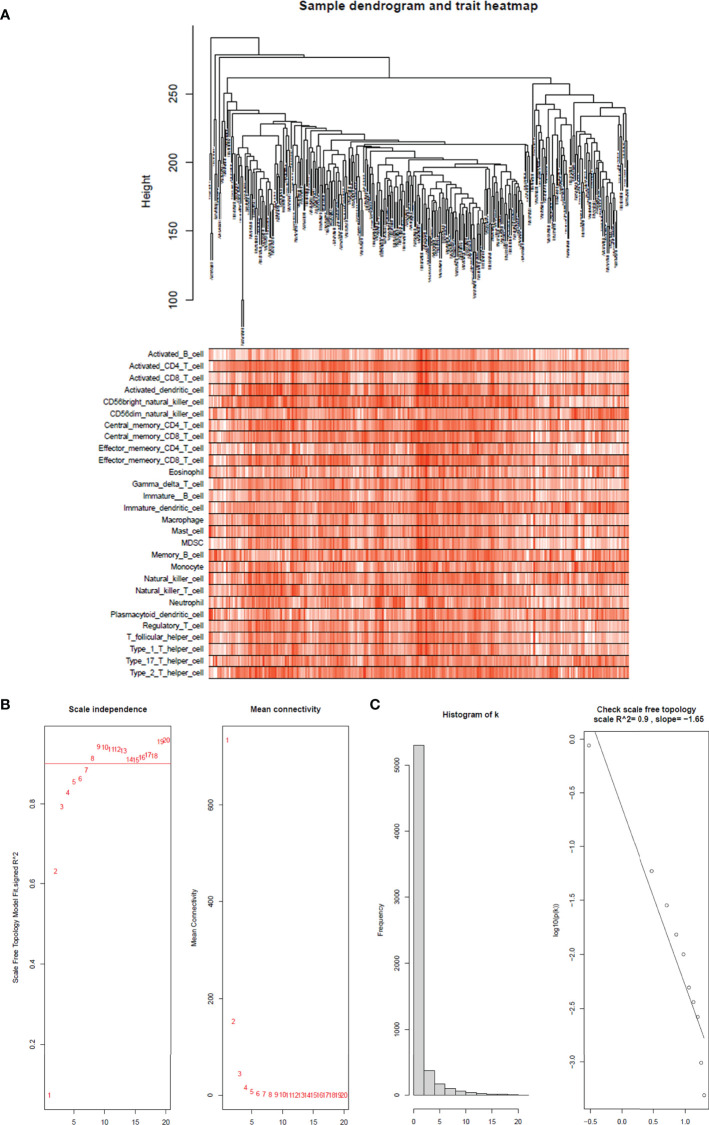
Sample tree and trait heatmap of 28 immune cells. **(A)** Sample dendrogram and trait heatmap of 28 immune cells. The clinical trait information is immunophenoscores of 28 infiltrated immune cells in each sample. **(B)** Analysis of the scale-free fit index for various soft-thresholding powers (β) and the mean connectivity for soft threshold powers. The soft-thresholding power in the WGCNA was determined on the basis of a scale-free R^2^ (R^2^ = 0.90). The left panel presents the relationship between the soft-threshold and scale-free R^2^. The right panel presents the relationship between the soft-threshold and mean connectivity. **(C)** Histogram of connectivity distribution when β = 8 and checking the scale-free topology when β = 8.

The top 25% of the gene expression of variance was screened by the quartile of gene expression level, and 609 genes were screened out to construct co-expressed gene networks and the sample dendrogram and trait heatmap are constructed (**Figure 4A**). In this study, the power of β = 8 was selected to ensure a scale-free network (**Figures 4B, C**) (scale-free R^2^ = 0.9, slope = −1.65).

**Figure 5:**
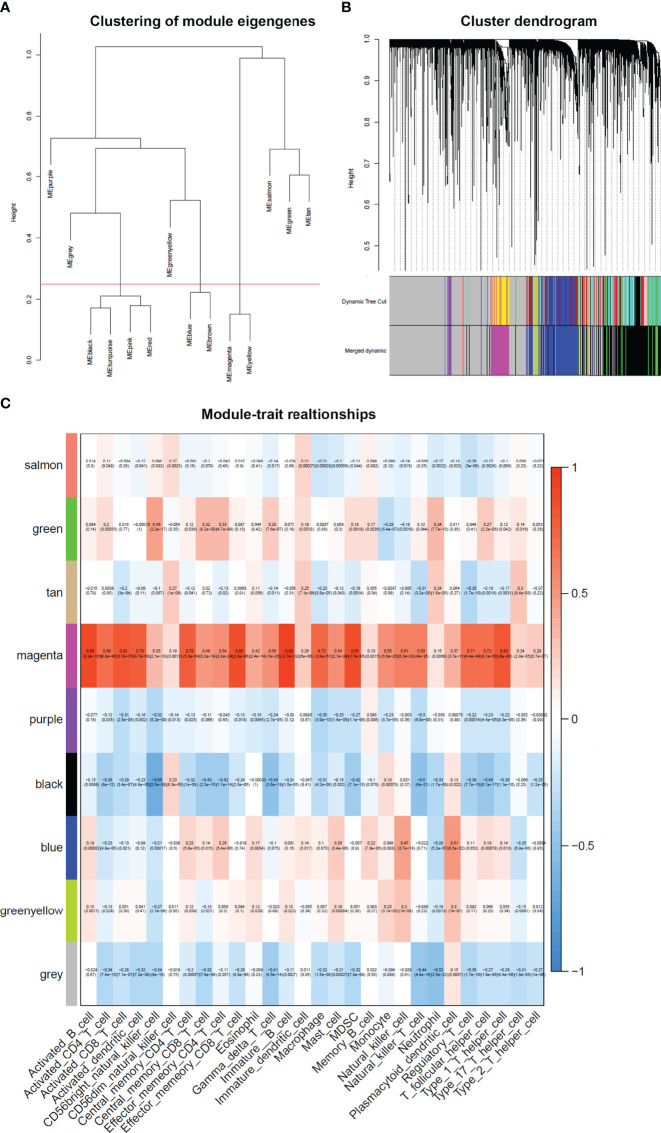
Weighted gene co-expression network in cervical cancer. **(A)** Clustering of module eigengenes. A cutline (0.25) was selected for module dendrogram and merged some modules according to dissimilarity of estimated module eigengenes. **(B)** A dendrogram of the differentially expressed genes clustered based on different metrics. Each branch in the figure represents one gene, and every color below represents one co-expression module. **(C)** A heatmap showing the correlation between the gene module and clinical traits. The correlation coefficient in each cell represented the correlation between gene module and the clinical traits. The magenta module showed the highest correlated with the infiltrated immune cells.

After merging the modules with the high similarity of feature genes in the gene cluster dendrogram through a cutline (0.25) (**Figure 5A**), nine modules were identified by the dynamic tree cut method. The clustering dendrograms of genes were shown in **Figure 5B**. A heatmap illustrating the correlation between immunophenoscores of infiltrated immune cells and key genes in the module was created (**Figure 5C**).

It was obviously that magenta module was the most correlated with immune infiltration in the heatmap. Hence, magenta module was selected as the clinical significant module for further analysis. We found that the correlation coefficient between magenta module and 11 immune infiltration cells was ≥ 0.7, which suggested strong correlation. This indicates that the genes in this module are most relevant to tumor OS. All 402 genes in magenta module were analyzed for survival of cervical cancer. Through univariate Cox regression survival analysis, 153 genes found to be significantly correlated with prognosis of cervical cancer were selected for further analysis.

### Screening the Key Genes and Survival Analysis

**Figure 6:**
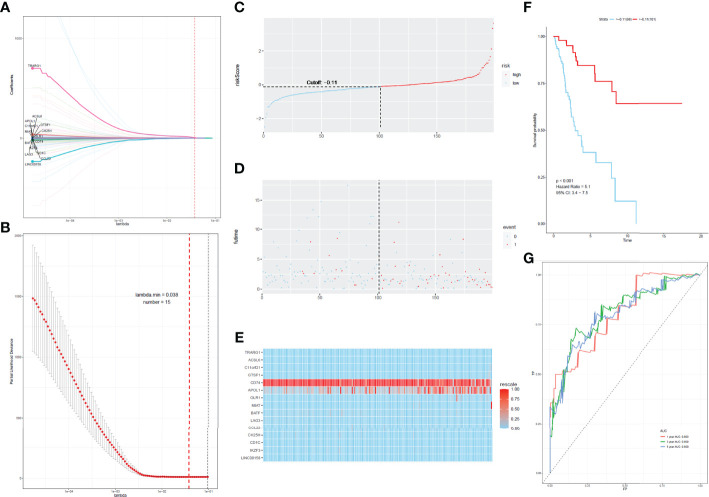
Construction of the prognostic classifier. **(A, B)** Determination of the number of factors by the LASSO analysis. **(C)** The distribution of risk score in test cohort. **(D)** The survival duration and status of patients in test cohort. **(E)** A heatmap of immune related genes in the classifier in test cohort. **(F)** Kaplan–Meier curve for patients with cervical cancer in test cohort. **(G)** ROC curve for cervical cancer in test cohort.

**Figure 7:**
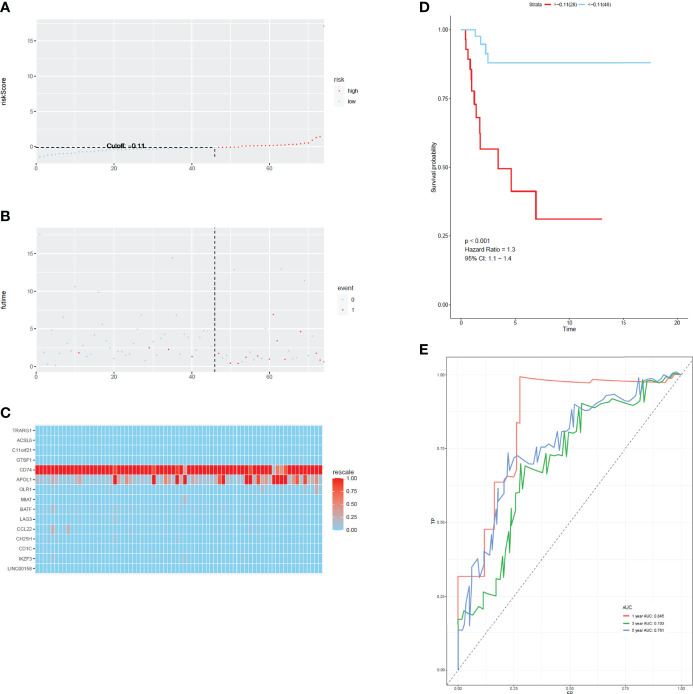
The distribution of time-dependent ROC curves and Kaplan–Meier survival based on the integrated classifier in the train cohort. **(A)** The distribution of risk score in train cohort. **(B)** The survival duration and status of patients in train cohort. **(C)** A heatmap of immune related genes in the classifier in train cohort. **(D)** Kaplan–Meier curve for patients with cervical cancer in train cohort. **(E)** ROC curve for cervical cancer in train cohort.

To develop a prognostic scoring model based on WGCNA, the 153 key genes were used to fit the LASSO Cox-PH model. Using parameter lambda (0.038) obtained upon performing 1000 cross-validations, a combination of 15 genes was obtained. These 15 genes were as follows: LAG3, CD74, CCL22, CH25H, OLR1, MIAT, BATF, IKZF3, TRARG, ACSL6, C11orf21, GTSF1, APOL1, CD1C, and LINC00158 (**Table 2** and **Figure 6**). Risk score of each sample in the training set was calculated. Then, the samples in the training cohort were divided into high- and low-risk groups according to median risk score. As demonstrated in **Figure 6**, the high-risk group patients in the training cohort have shorter OS time than the low-risk group (p < 0.001, HR: 5.1, 95% CI: 3.4–7.5), with an AUC of 0.803 in 1 year, 0.809 in 3 years, and 0.800 in 5 years. Next, the 15-gene signature was then verified by the validation cohort from TCGA. Consistently, the high-risk group patients in the validation cohort had worse prognosis than the low-risk group (p < 0.001, HR: 1.3, 95% CI: 1.1–1.4), and the AUC was 0.845 in 1 year, 0.703 in 3 years, and 0.761 in 5 years (**Figure 7**). All these suggested that the 15-gene signature associated with immune infiltration is able to predict prognosis in patients with cervical cancer.

**Table 2:** Fifteen genes from LASSO analysis.

Gene	HR	95% CI	P-value	Lasso Coefficient
TRARG1	660	(0.00027–1.6e+09)	0.39	4.7797999
ACSL6	3.4	(1.4–8.6)	0.008	0.653170468
C11orf21	1.3	(1.1–1.5)	0.00022	0.128185283
GTSF1	1.1	(1.0–1.2)	0.002	0.081672124
CD74	1.0	(1.0–1.0)	0.03	−8.18E−05
APOL1	1.0	(1.0–1.0)	0.016	0.001359139
OLR1	1.0	(1.0–1.0)	0.02	0.008057682
MIAT	1.0	(1.0–1.0)	5.00E−04	0.01207222
BATF	0.96	(0.93–1.0)	0.025	−0.006690799
LAG3	0.93	(0.86–0.99)	0.029	−0.012618
CCL22	0.90	(0.79–1.0)	0.073	−0.003652351
CH25H	0.88	(0.77–1.0)	0.053	−0.017915424
CD1C	0.78	(0.63–0.97)	0.027	−0.100839181
IKZF3	0.76	(0.63–0.93)	0.0061	−0.086985406
LINC00158	0.00062	(6.7e−07–0.58)	0.034	−0.708766884

### Immune Cell Subtypes Between High-Risk and Low-Risk Groups

**Figure 8:**
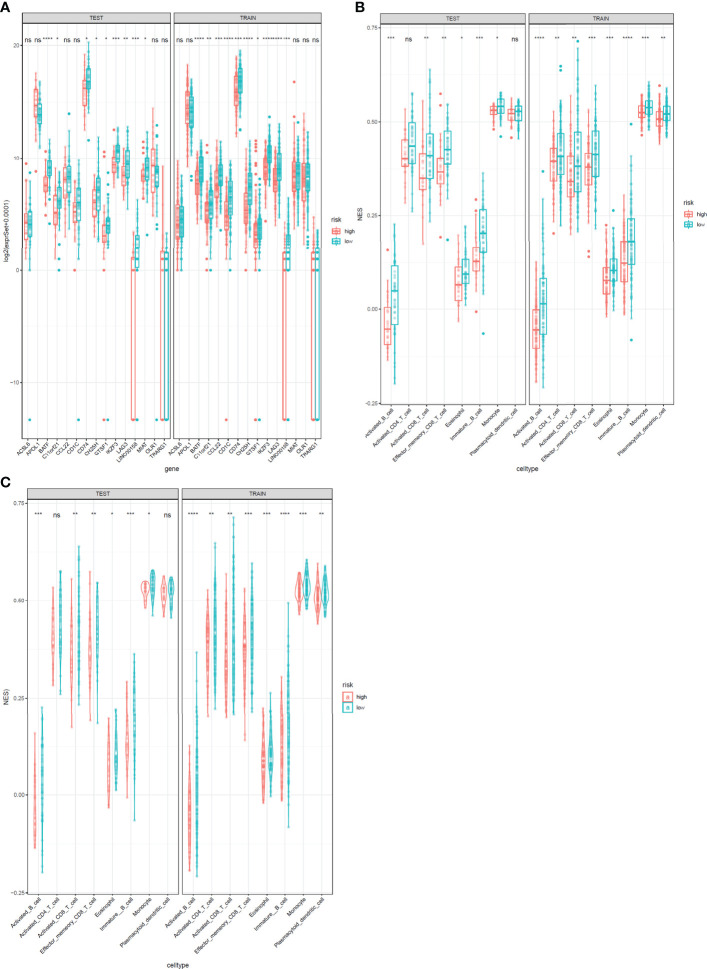
Expression profile of 15 genes and immunophenoscores of eight immune cell subtypes in test and train sets. **(A)** Expression profile of 15 genes. The test cohort is shown in the left, and the train cohort is shown in the right. **(B, C)** Immunophenoscores of eight infiltrated immune cells in high-risk and low-risk groups based on the integrated classifier. The test cohort is shown in the left, and the train cohort is shown in the right. *: P<0.05, **: P<0.01, ***: P<0.001****: P<0.0001, ns, no significance.

The expression levels of the 15 genes in test and validation cohorts are shown in **Figure 8A**. Different immune scores had differential OS in patients with cervical cancer. In both test cohorts and train cohorts, these eight immune cell subtypes (activated B cell, activated CD8 T cell, eosinophil, monocyte, activated CD4 T cell, effector memory CD8 T cell, immature B cell, and plasmacytoid dendritic cell) expressed differentially in high-risk and low-risk groups. (**Figures 8B, C**).

### GSEA Analysis

**Figure 9:**
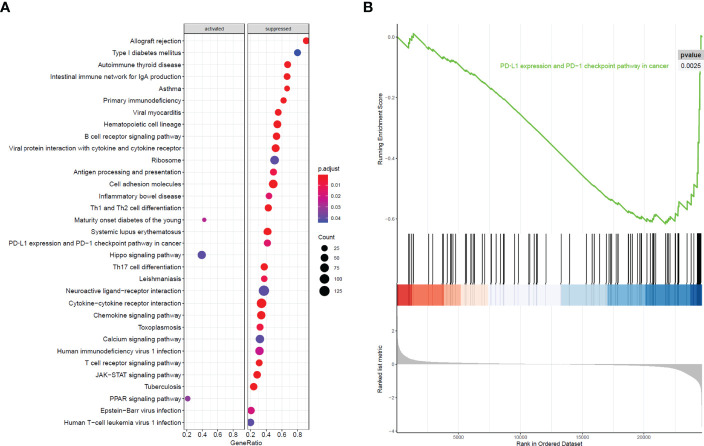
GSEA analysis and PD-L1 pathway. **(A)** GSEA analysis demonstrated 33 KEGG pathways associated with risk score in cervical cancer. **(B)** PD-L1 expression and PD-1 checkpoint pathway in cancer was one of the 33 pathways that significantly related with risk score.

All 304 primary cervical cancer samples were divided into high-risk group and low-risk group based on the 15-gene signature. To identify potential function of the 15 key genes, GSEA was conducted respectively to search “all gene sets” enriched in all 304 samples. GSEA showed 33 significant Kyoto Encyclopedia of Genes and Genomes (KEGG) pathway associated with risk score, including PD-L1 expression and PD-1 checkpoint pathway in cancer (**Figure 9** and **Table 3**). These results validated that the key genes in clinical significant module were mainly involved in the regulation of immune system. From GSEA, the 15-gene signature obviously participated in regulation of PD-1/PD-L1 pathway in cervical cancer.

**Table 3:** Significant KEGG pathways from GSEA analysis.

ID	Description	P-value	p-adjust	q-values
hsa04060	Cytokine-cytokine receptor interaction	3.29E−05	0.000668	0.000554
hsa04062	Chemokine signaling pathway	3.40E−05	0.000668	0.000554
hsa05152	Tuberculosis	3.42E−05	0.000668	0.000554
hsa04514	Cell adhesion molecules	3.45E−05	0.000668	0.000554
hsa05322	Systemic lupus erythematosus	3.49E−05	0.000668	0.000554
hsa04659	Th17 cell differentiation	3.52E−05	0.000668	0.000554
hsa04061	Viral protein interaction with cytokine and cytokine receptor	3.53E−05	0.000668	0.000554
hsa04660	T cell receptor signaling pathway	3.53E−05	0.000668	0.000554
hsa04640	Hematopoietic cell lineage	3.53E−05	0.000668	0.000554
hsa04658	Th1 and Th2 cell differentiation	3.54E−05	0.000668	0.000554
hsa04662	B cell receptor signaling pathway	3.56E−05	0.000668	0.000554
hsa05416	Viral myocarditis	3.63E−05	0.000668	0.000554
hsa05320	Autoimmune thyroid disease	3.64E−05	0.000668	0.000554
hsa04672	Intestinal immune network for IgA production	3.64E−05	0.000668	0.000554
hsa05340	Primary immunodeficiency	3.67E−05	0.000668	0.000554
hsa05330	Allograft rejection	3.67E−05	0.000668	0.000554
hsa05310	Asthma	3.70E−05	0.000668	0.000554
hsa04630	JAK-STAT signaling pathway	6.88E−05	0.001174	0.000974
hsa05145	Toxoplasmosis	0.000526	0.008506	0.007058
hsa04612	Antigen processing and presentation	0.000647	0.009924	0.008234
hsa05140	Leishmaniasis	0.000717	0.010486	0.008701
hsa05321	Inflammatory bowel disease	0.001157	0.01606	0.013326
hsa05235	PD-L1 expression and PD-1 checkpoint pathway in cancer	0.001203	0.01606	0.013326
hsa05169	Epstein-Barr virus infection	0.001524	0.01949	0.016172
hsa05170	Human immunodeficiency virus 1 infection	0.001867	0.022921	0.019019
hsa04950	Maturity onset diabetes of the young	0.002213	0.026135	0.021685
hsa03320	PPAR signaling pathway	0.002867	0.032598	0.027048
hsa04390	Hippo signaling pathway	0.003763	0.040506	0.03361
hsa03010	Ribosome	0.003912	0.040506	0.03361
hsa04080	Neuroactive ligand-receptor interaction	0.004057	0.040506	0.03361
hsa04020	Calcium signaling pathway	0.00416	0.040506	0.03361
hsa05166	Human T-cell leukemia virus 1 infection	0.004222	0.040506	0.03361
hsa04940	Type I diabetes mellitus	0.00475	0.044185	0.036663

## Discussion

Cervical cancer represents a major public health problem even in developed countries such as United States, with 14,000 new cases and 4,285 deaths estimated in 2022 (20). Increasing incidence of cervical adenocarcinoma and attenuation of earlier declines for cervical squamous cell carcinoma emphasize the importance of the need for improved therapeutic options to reduce the burden of cervical cancer (21). The most important risk factors affecting prognosis of cervical cancer are stage, status of the lymph nodes, tumor volume, depth of tumor invasion into the cervical stroma, and Lymphovascular space invasion (LVSI) (22). The 5-year OS decreased significantly with rising stages (23). A substantial percentage of patients with advanced cervical cancer will undergo recurrence and poor prognosis (2, 24), and the recurrence rate fluctuates from 10% to 74% (25). Tumor stage was one of the most pivotal factors related to recurrence: 10% for stage IB, 17% for stage IIA, 23% for stage IIB, 42% for stage III, and 74% for stage IVA (26). Prognosis of metastatic and recurrent cervical cancer was extremely poor with a median survival time of 12 months (27). Therefore, improved therapeutic options in management of cervical cancer, especially locally advanced cervical cancer is in urgent need.

High-risk subtypes of the human papilloma virus (HPV) are the cause of cervical cancer (28, 29). Viral oncoproteins E6 and E7 leads to dysregulation of p53 and Hypoxia-inducible factor-1 (HIF-1) alpha, thus affecting cell cycle proteins and VEGF expression (28–32). HPV is highly immunogenic and elicits immune responses in humans; thus, immune response might play important roles in carcinogenesis of cervical cancer. Incidence of cervical cancer was substantial declined in countries with high HPV vaccine coverage (33–35). Immunotherapy fights against tumor cells through activating endogenous immune response, which seems to switch on the new frontier of the anticancer treatment (36). Immunotherapy includes different approaches, such as active immunotherapy (vaccine), passive immunotherapy (adoptive cellular transfer, antibodies, and cytokines), and immunomodulation (cyclooxygenase 2 inhibitor).

Discovery of immune checkpoint such as CTLA-4 and PD-1 plays an indispensable role in the development of cancer immunotherapy. It was surprising that immune checkpoint inhibitors anti–CTLA-4 and anti–PD-1 displayed enormous success in solid tumors (37). Similar to other solid tumors, novel immunotherapeutic approaches, such as immune checkpoint inhibitors, have shown encouraging results in cervical cancer (12). Implementing immunotherapeutic approaches earlier in advanced cervical cancer would seem to be most appropriate. However, the objective response rate with anti–PD-1/PD-L1 monotherapy is hovering at 20%. Moreover, immune-related toxicities and severe adverse effects can occur during PD-1/PD-L1 blockade therapy (38). PD-1/PD-L1 blockade therapy does not demonstrate efficacy in almost 80% of patients with cervical cancer, suggesting that the potential mechanism of PD-1/PD-L1 in immunotherapy remains to be further clarified. Thus, new immune checkpoint inhibitors or comprehensive understanding of specific mechanism underlying PD-1/PD-L1 regulation in carcinogenesis is in urgent need.

Immune infiltration of cervical cancer determines the immune activation of tumor microenvironment and is related with clinical outcome of patients. In this study, immunophenoscore of 28 infiltrated immune cells in TCGA cervical cancer was calculated. Univariate Cox regression analysis showed that activated B cell, activated CD8 T cell, eosinophil, monocyte, activated CD4 T cell, effector memory CD8 T cell, immature B cell, and plasmacytoid dendritic cell were strongly associated with OS of cervical cancer. WGCNA revealed that magenta module was the most relevant module to immune infiltration. In total, we identified 10 kinds of infiltrated immune cells with strong correlation with magenta module. These were as follows: activated B cell (R = 0.88), activated CD8 T cell (R = 0.82), activated dendritic cell (R = 0.79), central memory CD4 T cell (R = 0.78), effector memory CD8 T cell (R = 0.85), immature B cell (R = 0.93), macrophage (R = 0.72), Myeloid-derived suppressor cells (MDSC) (R = 0.85), regulatory T cell (R = 0.71), T follicular helper cell (R = 0.72), and type 1 T helper cell (R = 0.83).

By the use of WGCNA, magenta module showed the highest correlation with the immune infiltration of cervical cancer. The magenta module contained 609 immune-related genes, and 153 genes were picked up for further analysis after taking intersection of prognosis and immune infiltration. Through LASSO analysis, 15 immune-related genes (LAG3, CD74, CCL22, CH25H, OLR1, MIAT, BATF, IKZF3, TRARG, ACSL6, C11orf21, GTSF1, APOL1, CD1C, and LINC00158) were included in prognostic classifier. In addition to CTLA-4– and PD-1/PD-L1–targeted cancer immunotherapy, LAG3 (lymphocyte activation gene-3, CD223) is the third clinically targeted inhibitory receptor (39). CD74 (invariant chain) plays a dispensable role in process of immune systems that it participates in antigen presentation, B-cell differentiation, and inflammatory signaling. CD74 has the potential to be a therapeutic target in cancer and autoimmune disease (40). The chemokine CCL22 promoted regulatory T cell communication with dendritic cells to control immunity and was associated with poor prognosis (41, 42). CH25H produces 25-hydroxycholesterol, which inhibited tumor-derived extracellular vesicles uptake and correlated with prognosis in patients with melanoma (43). OLR1 (oxidized low-density lipoprotein (LDL) receptor 1) was a possible link between obesity, dyslipidemia, and cancer. OLR1 played carcinogenic role by activating Nuclear factor kappa B (NF-κB) pathway to promote proliferation and migration and to inhibit apoptosis and *de novo* lipogenesis (44). MIAT Long non-coding RNAs (lncRNA) was overexpressed in a number of malignancies and caused poor prognosis (45–47). Basic leucine zipper transcription factor, ATF-like (BATF) was an important transcription factor regulating differentiation of early effector CD8^+^ T cells (48) and was a prognostic indicator for patients with colon cancer (49). IKZF3 promoted growth of multiple tumors to cause poor prognosis (50).

Immune infiltration played important roles in the survival of cervical cancer. In this study, we identified 15 immune infiltration associated genes in cervical cancer and built a prognostic signature. Immune scores depended on the expression of these 15 genes and were associated with the survival of cervical cancer. High immune scores meant good prognosis. GSEA analysis showed that the 15-gene prognostic signature was obviously associated with PD-L1 expression and PD-1 checkpoint pathway in cancer. The prognostic signature could provide basis for potential immunotherapy in the future. Similarly, other researchers also constructed prognostic signatures for cervical cancer based on immune-related genes (51, 52). Compared with our study, although these studies use different types and different quantity of genes, all the signatures can well predict the prognosis of cervical cancer. However, the study has several limitations. First, no *in vitro* or *in vivo* molecular experiment was performed to verify our analysis. Second, our study was a retrospective study. Thus, prospective study is in need to validate the findings of our study in the future.

## Conclusion

In conclusion, we successfully constructed a 15-gene prognostic signature with powerful predictive function. Differences in the OS of high- and low-risk groups are implicated in immune infiltration, tumor microenvironment, PD-L1 expression, and PD-1 checkpoint pathway. These findings revealed the underlying mechanism of immunotherapy and provided basis for cervical cancer pathogenesis and clinical treatment.

## Data Availability Statement

The original contributions presented in the study are included in the article/supplementary material. Further inquiries can be directed to the corresponding authors.

## Author Contributions

RS is responsible for experimental design. CWJ and HB are responsible for instrument operation. JX is responsible for data analysis. LZ and CJJ are for providing overall ideas. All authors contributed to the article and approved the submitted version.

## Funding

This work was supported by the National Natural Science Foundation of China (grant no. 82002736 to CJJ) and the clinical characteristic medical technology cultivation plan of Shanghai General Hospital (no. 02.DY12.06.22.07 to LZ and no. 02.DY12.06.22.12 to JX).

## Conflict of Interest

The authors declare that the research was conducted in the absence of any commercial or financial relationships that could be construed as a potential conflict of interest.

## Publisher’s Note

All claims expressed in this article are solely those of the authors and do not necessarily represent those of their affiliated organizations, or those of the publisher, the editors and the reviewers. Any product that may be evaluated in this article, or claim that may be made by its manufacturer, is not guaranteed or endorsed by the publisher.
